# Minimal Detectable Changes in the Five Times Sit-to-Stand Test in Older Japanese Adults with Sarcopenia Requiring Long-Term Care

**DOI:** 10.3390/medicina59112019

**Published:** 2023-11-16

**Authors:** Lu Yin, Yohei Sawaya, Ryo Sato, Takahiro Shiba, Tamaki Hirose, Ko Onoda, Tomohiko Urano

**Affiliations:** 1 Nishinasuno General Home Care Center, Department of Day Rehabilitation, Care Facility for the Elderly “Maronie-en”, 533-11, Iguchi, Nasushiobara 329-2763, Tochigi, Japan; 20s3008@g.iuhw.ac.jp (L.Y.); sawaya@iuhw.ac.jp (Y.S.); 19s1095@g.iuhw.ac.jp (R.S.); t-shiba@iuhw.ac.jp (T.S.); n-tamaki@iuhw.ac.jp (T.H.); 2 Department of Physical Therapy, School of Health Sciences, International University of Health and Welfare, 2600-1 Kitakanemaru, Otawara 324-8501, Tochigi, Japan; ko_onoda@iuhw.ac.jp; 3 Department of Geriatric Medicine, School of Medicine, International University of Health and Welfare, 4-3 Kozunomori, Narita 286-8686, Chiba, Japan

**Keywords:** elderly, Japan, long-term care, reliability, sarcopenia

## Abstract

*Background and Objectives*: Although the importance of sarcopenia control has been suggested, there are no minimal detectable change (MDC) studies of older adults with sarcopenia, to our knowledge, and the criteria for determining the effectiveness of interventions are unknown. The purpose of this study was to calculate the MDC in the five times sit-to-stand test (FTSST) in older Japanese adults with sarcopenia and use it as an index to determine the effectiveness of future interventions. *Materials and Methods*: This was a cross-sectional study conducted in January and February 2023. The participants of this study were older Japanese adults using daycare rehabilitation. Thirty-eight participants performed the FTSST twice a week. Grip strength, walking speed, and skeletal muscle mass were measured to determine the presence of sarcopenia. The diagnosis of sarcopenia was defined as low skeletal muscle mass and low muscle strength and/or low physical function, based on the Asian Working Group for Sarcopenia 2019 diagnostic criteria. Participants were further classified as sarcopenic or non-sarcopenic. Intraclass correlation coefficients (ICCs) and MDCs were calculated for the overall, sarcopenia, and non-sarcopenia groups using the two FTSST measures. The average and difference of the two variables were used to calculate the MDC. *Results*: Overall, the ICC (1,1) was 0.94, MDC was 2.87 s, and MDC% was 23.3%. The sarcopenia group had an ICC (1,1) of 0.93, MDC of 3.12 s, and MDC% of 24.0%. The non-sarcopenia group had an ICC (1,1) of 0.95, MDC of 2.25 s, and MDC% of 19.2%. *Conclusions*: Despite the limitation of the data being only from this study population, we found that a change of ≥3.12 s or ≥24.0% in the FTSST of older adults with sarcopenia was clinically meaningful and may help to determine the effectiveness of sarcopenia treatment. The improvement or decline in older Japanese adults with sarcopenia should be determined by changes in the FTSST over a longer period of time than that for other conditions.

## 1. Introduction

According to the United Nations, one in six people globally will be 60 years or older by 2030, and by 2050, the global population over 60 years will more than double (2.1 billion people) [1]. Japan has the longest life expectancy in the world; as of 2023, the average life expectancy in Japan is 87.09 years for females and 81.05 years for males [2]. However, there is a disparity of approximately 10 years between average life expectancy and healthy life expectancy [3], which needs to be resolved. In order to extend healthy life expectancy, it is necessary to evaluate characteristic geriatric conditions that may be risk factors for shorter healthy life expectancy in older adults.

The global aging population has stimulated research on sarcopenia. Sarcopenia is the age-related decline in skeletal muscle mass and muscle strength and/or physical function [4]. Sarcopenia is known to cause a decline in physical function [5], limitation of daily activities [6], increased risk of falls and fractures [7,8], and other complications, resulting in social and economic burdens [9], such as shortened healthy life expectancy and increased medical costs. Furthermore, the risk of death in patients with sarcopenia has been reported to be significantly higher than in patients without sarcopenia [10].

As sarcopenia affects the healthy life expectancy of older adults, an evaluation and understanding of sarcopenia is essential. Sarcopenia is coded in the International Classification of Diseases 10th Revision and recognized as an independent disease. Several groups have proposed definitions regarding diagnostic criteria for sarcopenia. Representative examples of sarcopenia diagnostic criteria include the European Working Group on Sarcopenia in Older People (EWGSOP) in 2010, the Asian Working Group for Sarcopenia (AWGS) in 2014, and the EWGOSP2 in 2018 [11]. Recently, the AWGS 2019 was published, and many Asian countries, including Japan, use the AWGS 2019 criteria to diagnose sarcopenia [4].

The reported prevalence of sarcopenia in the Japanese population is 9.9% in community-dwelling older adults [12], 53.8% in patients admitted to rehabilitation wards [13], 53.5% in stroke patients [14], and 45.2–60.2% in older adults requiring nursing care [15,16]. Therefore, assessment and intervention for sarcopenia are essential for older adults who are eligible for rehabilitation.

The five times sit-to-stand test (FTSST) is a test that measures the time required to perform chair-rise movements as quickly as possible [17]. The FTSST has been adopted by Asian and European countries as a standard assessment method for diagnosing sarcopenia [4,11], and it has been reported that a longer FTSST time is associated with abnormal balance and gait patterns and an increased risk of falls in older adults [18,19]. Previous studies on the reliability of the FTSST have reported intraclass correlation coefficients (ICCs) of 0.8 or higher, indicating that the reliability of the FTSST is very high [20,21,22,23,24,25]. ICC is a measure of relative reliability and provides information about the degree of agreement between multiple measurements.

Absolute reliability, on the other hand, is a measure for determining the type of error or bias in a measurement. Absolute reliability can be estimated by the standard error of measurement (SEM) and the minimal detectable change (MDC) [26]. The MDC represents the limit of measurement error, and changes above the MDC are considered “true change” [27]. Measurement error affects measurement accuracy, and if the measurement error is too large, clinical application becomes difficult due to accuracy issues. The MDC is a clinically important measure for determining the effect of exercise in longitudinal interventions. It can also provide evidence for clinicians to determine whether different measurements are beneficial. To the best of our knowledge, as criteria for sarcopenia have been proposed in recent years, no study has calculated the MDC of the FTSST separately for patients with and without sarcopenia.

The purpose of this study was to calculate the MDC of the FTSST in older Japanese adults with sarcopenia requiring long-term care and to use it as an index to determine the effectiveness of future interventions. Since the MDC is influenced by population characteristics, region, age, and ethnicity [28,29,30], the calculation of the MDC in the FTSST targeting older adults with sarcopenia who require long-term care in an aging Japanese population is of great significance in determining exercise effectiveness. We hypothesized that older adults with sarcopenia would spend more time performing the FTSST and that the MDC would be greater.

## 2. Materials and Methods

### 2.1. Study Design

This single-center, cross-sectional study was conducted between January and February 2023. The study was approved by the Ethics Review Board (approval number: 21-Io-22-2) on 15 March 2023 and conducted in accordance with the guidelines of the Declaration of Helsinki. All participants and their family members signed an informed consent form.

### 2.2. Participants

**Figure 1:**
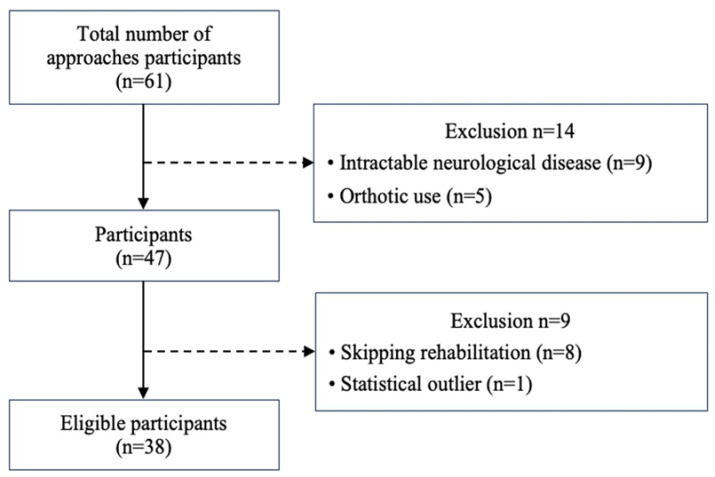
Flowchart for participant enrollment.

The participants of this study were 61 older adults aged 65 years or older who used daycare rehabilitation from January 2023 to February 2023, were determined to need support levels 1–2 and care levels 1–2 by Japanese long-term care certification, and were able to stand up and walk independently. Figure 1 shows the flowchart of the study participants. A total of 61 older Japanese adults participated in the study. To ensure the reproducibility of the FTSST measurements, nine participants with intractable neurological disease (seven with Parkinson’s disease, one with progressive supranuclear palsy, and one with spinocerebellar degeneration) and five with orthotic use were excluded, leaving 47 participants for analysis. Of the participants analyzed, eight were excluded for missing rehabilitation sessions, and one because the difference between the day 1 and day 2 measurements was a statistical outlier. Therefore, 38 participants (20 males and 18 females, mean age 79.9 ± 6.8 years) were included in the final analysis.

In Japan, medical insurance and long-term care insurance are administered by the national system. In order to receive long-term care insurance services, a person must be certified as requiring long-term care or support. The certification for care and support required is determined by the attending doctor, a computer system, and trained municipal officials, based on the person’s physical and mental condition. There are seven levels of long-term care that require certificates: support levels 1 (least disabled) and 2 and care need levels 1 to 5 (most disabled) [31]. The daycare facility provides rehabilitation services, including transportation services, in accordance with the long-term care insurance system.

### 2.3. Medical Record (Basic Attributes and Clinical Characteristics)

Medical records obtained from the doctors were consulted. Basic attributes and clinical characteristics were obtained from medical records. The medical diagnosis was investigated as a clinical characteristic. The medical diagnoses of participants were obtained from the diagnosis by the primary doctor who prescribed daycare and ambulatory rehabilitation for older adults in the long-term care insurance system. The diagnoses were confirmed as cerebrovascular disease, circulatory disease, cancer, diabetes mellitus, fracture, spine disease, osteoarthritis, or renal failure.

### 2.4. FTSST

Participants performed the FTSST twice during the week, each on a different day; the interval between days 1 and 2 ranged from 1 to 7 days, depending on the day of the rehabilitation plan. The height of the chair used for the FTSST was 41 cm, which was the same as that used in previous studies [32]. In the starting posture, the participants sat shallowly in a chair with both lower limbs shoulder-width apart and arms crossed in front of the chest. Following the measurer’s “ready” signal, the participant moved to a standing posture with the torso and both knee joints fully extended, and immediately returned to a sitting posture upon the measurer’s “start” signal. This was repeated five times, and the time from the fifth rise to the completion of seating was recorded. All tests were performed by a single physical therapist, and all participants underwent actual measurements after one practice session. The physical therapist verbally instructed the participant to “fully extend both knees when standing up” and “place the buttocks on the chair when sitting down”. All FTSST measurements were performed before exercise therapy during the day rehabilitation program to ensure that the participants were not fatigued at the time of measurement.

### 2.5. Methods for Measuring Grip Strength, Gait, Skeletal Muscle Mass, and Height

Grip strength was measured using a grip-strength meter (Grip D-TKK5401; Takei Scientific Instruments Co., Ltd., Tokyo, Japan). Grip strength was measured with participants in a seated position, not leaning on their backs. The left and right sides were measured twice each, and the highest value was recorded as the representative value. Walking speed was calculated as the usual walking speed between 3 m and 8 m (5 m) on an 11 m walking path marked at 3 m and 8 m. Walking aids were typically used. A manual stopwatch was used for the measurements. Skeletal muscle mass was measured by bioelectrical impedance analysis (BIA) using a body composition analyzer (TANITA MC780-A; Tanita, Tokyo, Japan) in the standing position. This is a multi-frequency body composition instrument that measures at three frequencies. Skeletal muscle mass index (SMI) was calculated by dividing limb muscle mass by the height squared.

The diagnosis of sarcopenia was based on the diagnostic criteria of the AWGS 2019. Grip strength was used to determine muscle strength, walking speed to determine physical function, and SMI to determine the skeletal muscle mass using the BIA method [4]. The diagnosis included cut-off values of muscle weakness (grip strength: <28 kg in males and <18 kg in females), physical dysfunction (walking speed: <1 m/s), and skeletal muscle mass loss (SMI: <7.0 kg/m^2^ in males and <5.7 kg/m^2^ in females). Sarcopenia was defined as low skeletal muscle mass plus low muscle strength and/or low physical function. Participants were classified as sarcopenic or non-sarcopenic.

### 2.6. Sample Size and Study Power

In a similar previous study calculating MDC, the required sample size was calculated according to the suggestion of Walter et al. [33,34]. As a result, the required sample size was at least 11 participants. Of the 38 participants in this study, 18 were placed in the sarcopenia group and 20 in the non-sarcopenia group; our sample size is larger than the 11 participants in the previous study, and it was considered adequate.

### 2.7. Statistical Analyses

Comparisons between groups with and without sarcopenia were performed using the Fisher’s exact probability test (for sex and main diagnosis), unpaired *t*-test (for age, height, body weight, and body mass index [BMI]), and Mann–Whitney test (for certification level). Age, height, weight, BMI, and FTSST were statistically analyzed after confirming normality using the Kolmogorov–Smirnov and Shapiro–Wilk tests. The Grubbs–Smirnov test was performed to identify outliers in the difference between the day 1 and day 2 measurements. Next, to determine the absolute reliability of the FTSST, a Bland–Altman analysis was performed on days 1 and 2, and a Bland–Altman plot was created with the mean of the two measurements on the *x*-axis and the difference between the two measurements on the *y*-axis to calculate the MDC. The MDC indicates the limit range where the amount of change between two measurements obtained by repeated measurements, such as retests, is due to measurement error. Firstly, the SEM was calculated using the following formula [35]:

(1) $$\mathrm{SEM}=\mathrm{Standard}\;\mathrm{deviation}/\sqrt{2}$$

Next, the MDC was calculated. The formulae used in this study were as follows [36,37]:

(2) $$\mathrm{MDC}=1.96\;\times \;\sqrt{2}\times \mathrm{SEM}$$

and [38,39]


(3) MDC %= (MDC/mean)× 100

We employed (SD/$\sqrt{2}$) as the calculation method for SEM; there are several methods for calculating SEM, but only negligible differences have been reported [40,41]. The standard deviation and average were calculated from the difference between measurements on days 1 and 2, and the ICC was calculated (1,1). Kappa coefficients were calculated to determine the agreement between sarcopenia diagnosed using gait speed and that diagnosed using the FTSST. SPSS version 26 software for Mac (IBM Corp., Armonk, NY, USA) was used for statistical analysis. The statistical significance level was set at 5%.

## 3. Results

Table 1 shows a comparison of sarcopenia between the groups of participants. Of the 38 participants included in the final analysis, 18 were classified as sarcopenic and 20 as non-sarcopenic. Comparison between the groups with and without sarcopenia showed significant differences in BMI, the level of care required, and circulatory disease. Table 2 shows measurement values in the FTSST between day 1 and day 2. The respective FTSST readings were 12.4 ± 4.3 on day 1, 12.2 ± 4.2 on day 2, and 12.3 ± 4.2 on average for the overall group; 13.0 ± 4.2 on day 1, 13.1 ± 4.3 on day 2, and 13.0 ± 4.2 on average for the sarcopenia group; and 12.1 ± 4.6 on day 1, 11.3 ± 4.1 on day 2, and 11.7 ± 4.3 on average for the non-sarcopenia group.

**Figure 2:**
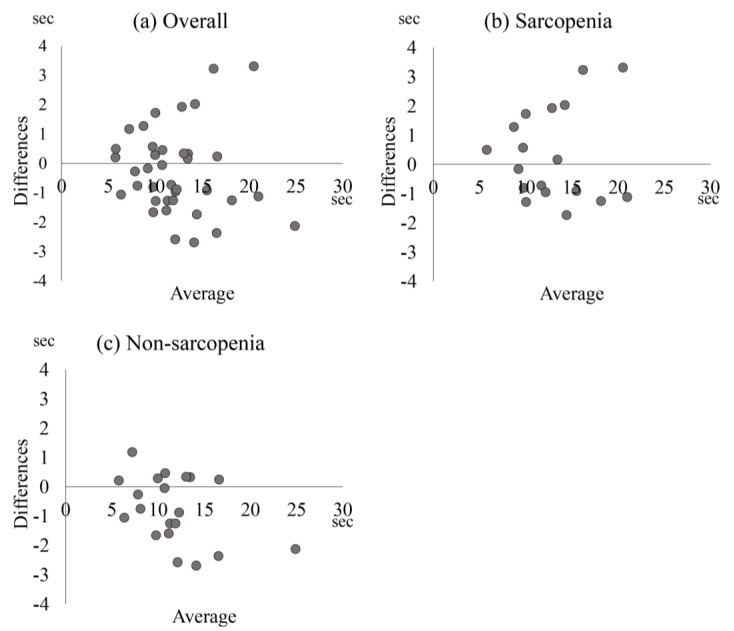
Bland–Altman plot of the five times sit-to-stand test values. Bland–Altman plot shows the five times sit-to-stand test values for (**a**) overall population, (**b**) sarcopenia, and (**c**) non-sarcopenia. The vertical axis represents measurement differences between days 1 and 2. The horizontal axis represents the average value of days 1 and 2.

Table 3 and Figure 2 show the results of the Bland–Altman analysis and the MDC of the FTSST. The ICCs (1,1) and 95% confidence intervals were 0.94 (0.89–0.97) overall, 0.93 (0.83–0.97) with sarcopenia, and 0.95 (0.88–0.98) without sarcopenia. The MDC was 2.87 s overall, 3.12 s with sarcopenia, and 2.25 s without sarcopenia. The MDC% was 23.3% overall, 24.0% with sarcopenia, and 19.2% without sarcopenia. The SEM was 1.77 overall, 1.12 with sarcopenia, and 0.81 without sarcopenia.

We also confirmed the agreement of physical function between sarcopenia diagnosed by gait speed and sarcopenia diagnosed by FTSST. The results showed that the kappa coefficient was 0.84, indicating almost perfect agreement.

## 4. Discussion

Sarcopenia is closely related to physical performance, quality of life, and health care demand [5,6,7,8,9], and is one of the main topics of interest in geriatric medicine. In recent years, there have been numerous intervention studies on sarcopenia, but the criteria for determining improvement are unclear [42,43]. This study revealed an MDC of 3.12 s and MDC% of 24.0% for the FTSST in participants with sarcopenia. A change larger than the MDC represents a real change, not a random difference. To our knowledge, this is the first study to calculate clinically meaningful changes only in people with sarcopenia and may contribute to determining the effectiveness of future interventions for sarcopenia. Especially in a hyper-aged society, this has clinical relevance as a clear criterion for determining the effectiveness of interventions for older adults who need geriatric medicine and patient care.

The ICC (1,1) of the FTSST in this study was greater than 0.9 in all groups. The ICCs (1,1) of the FTSST in previous studies were 0.867–0.905 for patients with hip osteoarthritis (HOA), 0.970 for patients with chronic obstructive pulmonary disease, and 0.962 for patients with spinal cord injury (SCI) [22,23,24]. Therefore, the FTSST, an evaluation of lower limb muscle strength, has excellent reliability, even in participants with sarcopenia, which affects physical performance and activity in daily life. In recent years, an increasing number of studies have used FTSST as a longitudinal change outcome [44,45,46].

As MDC values differ by population and disease, the results of this study and the disease-specific MDC values from previous studies are discussed here. The MDC of the FTSST of the sarcopenia group in this study was 3.12 s, whereas in previous studies, it was 1.47–2.31 s in patients with HOA, 2.93 s in patients with SCI, and 1.6 s in healthy older adults [20,22,24]. Thus, as hypothesized, the sarcopenia group had a greater MDC compared with other conditions. The results in the sarcopenia group in our study may have been influenced by the fact that variations in physical performance in older adults are related to lower limb skeletal muscle strength and aging [47,48,49] and that the participants in this study were older than those in previous studies, which may have increased both execution time and MDC. In fact, in this study, because the sarcopenia group had a longer execution time and MDC than the non-sarcopenia group, the potentials of the populations may differ. This suggests that when determining the effectiveness of exercise in the FTSST, a reduction of 3.12 s, a longer time, should be considered as an improvement in older adults with sarcopenia compared to those without sarcopenia.

The MDC% of sarcopenia in the FTSST test was 24.0%. In previous studies, it was 15.2–21.4% in participants with HOA, 18.7% in participants with SCI, and 16.1% in healthy older adults [20,22,24]. Another study showed an MDC% of 25.9% for participants with cardiac disease [50]. Although the MDC% of sarcopenia in this study was greater than that of other conditions evaluated in previous studies, the trend by which older adults with sarcopenia could be judged to improve or deteriorate with changes in the FTSST over a longer period of time was similar for both the MDC and MDC%. In previous studies, the MDC% of grip strength, an endpoint of sarcopenia, was 13.8% in participants with subacute stroke [51], 17.4% in participants with heart disease [50], and 14.5% in participants with diabetes [52]. Further studies showed an MDC% for gait speed of 19.6% [39] in participants with COPD, 14.3% in participants with heart disease [50], and 24.4–28.5% in participants with dementia-related body conditions [38]. The MDC% of grip strength, gait speed, and FTSST, which are indices to evaluate sarcopenia, have shown values of approximately 20%. According to the Guidelines for the Treatment of Sarcopenia [4,11], grip strength and walking speed are also recommended to assess muscle strength and physical function. In the future, it will be necessary to calculate the MDC of other indices to search for a more sensitive evaluation method for determining the effect of exercise on sarcopenia.

In this study, we also confirmed the agreement between sarcopenia diagnosed using gait speed and that diagnosed using the FTSST with regard to physical function. The result showed that the kappa coefficient was 0.84, indicating almost perfect agreement [53]. The FTSST is reproducible as an evaluation method for the diagnosis of sarcopenia, even in older adults requiring nursing care. Generally, gait speed is used to evaluate physical function when diagnosing sarcopenia. However, walking speed is often not feasible in environments such as small clinical spaces or home rehabilitation. The advantage of the FTSST is that it can be performed anywhere on a chair. The results of this study suggest that the FTSST should be considered as an evaluation of physical function in the diagnosis of older Japanese adults with sarcopenia who require long-term care.

This study had several limitations. The results of this study were based on a small sample size from the same institution, and a study with a larger sample size is required to support the generalization of our results. However, the method used to calculate the sample size based on previous studies indicated that our study had an appropriate sample size. Second, this study was conducted on older adults requiring nursing care who presented with several chronic diseases, and caution should be taken when adapting the study to older adults who do not require nursing care. It is difficult to completely exclude disease-related factors in Japan, where the rate of comorbidity of multiple diseases among older adults is reported to be 62.8% [54]. Third, the MDCs of other sarcopenia diagnostic items (grip strength and gait speed) were not calculated in this study. The MDCs of sarcopenia diagnostic items should be calculated in various subgroups of older adults to clarify the criteria for clinical efficacy. Despite these limitations, our study may help determine the effectiveness of sarcopenia treatment research in future interventions. In Japan’s super-aging society, determining the effectiveness of interventions for older adults requiring nursing care may be helpful.

## 5. Conclusions

In the present study, the MDC, one of the indices for determining the effectiveness of interventions, was calculated for the FTSST in older adults with sarcopenia. The MDC of the FTSST in older adults with sarcopenia was 3.12 s and 24.0%. This suggests that a change in FTSST measurements greater than 3.12 s or 24.0% may indicate a meaningful real change. The results of this study may assist in the future treatment of sarcopenia.

## Figures and Tables

**Table 1:** Clinical characteristics of the study participants.

	Overall (*n* = 38)	Sarcopenia (*n* = 18)	Non-Sarcopenia (*n* = 20)	*p*-Value
Age (years)	79.1 ± 7.6	80.9 ± 7.1	79.0 ± 6.6	0.402
Males	20 (52.6%)	11 (61.1%)	9 (45%)	0.481
Height (cm)	159.1 ± 9.5	158.4 ± 7.3	157.0 ± 10.1	0.642
Body weight (kg)	57.0 ± 8.8	54.9 ± 8.6	58.8 ± 8.8	0.183
BMI (kg/m^2^)	23.1 ± 2.9	21.9 ± 2.5	24.1 ± 2.9	0.019 *
Certification levels (1–4) ^1^	2 (1–3)	3 (1.75–3)	1 (1–2)	0.006 *
Main diagnosis				
Cerebrovascular disease	18 (47%)	9 (50%)	9 (45%)	0.338
Circulatory disease	10 (26%)	7 (38.9%)	3 (15%)	0.043 **
Cancer	4 (11%)	1 (0.1%)	3 (15%)	0.911
Diabetes mellitus	6 (16%)	3 (16.7%)	3 (15%)	0.888
Fracture	8 (21%)	3 (16.7%)	5 (25%)	0.529
Spine disease	10 (26%)	4 (22.2%)	6 (30%)	0.587
Osteoarthritis	2 (5%)	0 (0%)	2 (10%)	0.126
Renal failure	4 (11%)	1 (0.1%)	3 (15%)	0.911

Values are presented as mean ± standard deviation. Certification levels are classified into four levels (support levels 1–2 and care levels 1–2). ^1^ Median (25 percentile–75 percentile). * Unpaired *t*-test/Mann–Whitney U test of sarcopenia vs. non-sarcopenia, *p* < 0.05. ** Fisher’s exact test of sarcopenia vs. non-sarcopenia. BMI, body mass index.

**Table 2:** Measurement values in the five times sit-to-stand test between day 1 and day 2.

	The Five Times Sit-to-Stand Test (s)		
	Day 1	Day 2	Average
Overall (*n* = 38)	12.4 ± 4.3	12.2 ± 4.2	12.3 ± 4.2
Sarcopenia (*n* = 18)	13.0 ± 4.2	13.1 ± 4.3	13.0 ± 4.2
Non-sarcopenia (*n* = 20)	12.1 ± 4.6	11.3 ± 4.1	11.7 ± 4.3

Values are presented as mean ± standard deviation.

**Table 3:** Results of the minimum detectable change for the five times sit-to-stand test measurements.

	ICC (1,1)	95% CI	SEM	MDC	% MDC
Overall (*n* = 38)	0.94	0.89–0.97	1.77	2.87	23.3
Sarcopenia (*n* = 18)	0.93	0.83–0.97	1.12	3.12	24.0
Non-sarcopenia (*n* = 20)	0.95	0.88–0.98	0.81	2.25	19.2

Values are presented as mean ± standard deviation. CI: confidence interval; ICC: intraclass correlation coefficient; MDC: minimal detectable change. SEM: standard error of the mean.

## Data Availability

Data are contained within the article.
